# LINC00460 Stimulates the Proliferation of Vascular Endothelial Cells by Downregulating miRNA-24-3p

**DOI:** 10.1155/2022/2524156

**Published:** 2022-02-18

**Authors:** Ruofei Jia, Xingxing Yuan, Chengzhi Yang, Jing Han, Xiaojing Cao, Zheng Qin, Jing Nan, Zening Jin

**Affiliations:** ^1^Department of Cardiology, Beijing Tiantan Hospital, Capital Medical University, Beijing, China; ^2^Department of Clinical Laboratory Center, Beijing Youan Hospital, Capital Medical University, Beijing, China

## Abstract

**Objective:**

To clarify the effect of LINC00460 on mediating the proliferative ability of vascular endothelial cells (ECs) by targeting microRNA-24-3p (miRNA-24-3p), thus influencing the progression of atherosclerotic diseases.

**Methods:**

Relative levels of LINC00460 and miRNA-24-3p in ECs induced with different doses of ox-LDL (oxidized low density lipoprotein) for different time points were determined by quantitative real-time polymerase chain reaction (qRT-PCR). Influences of LINC00460 and miRNA-24-3p on the viability of ECs were assessed by Cell Counting Kit-8 (CCK-8) and 5-ethynyl-2′-deoxyuridine (EdU) assay. Through dual-luciferase reporter gene assay, the binding between LINC00460 and miRNA-24-3p was evaluated. At last, rescue experiments were performed to identify the function of the LINC00460/miRNA-24-3p axis in regulating the proliferative ability of ECs.

**Results:**

LINC00460 was upregulated after ox-LDL treatment in a dose- and time-dependent manner. Viability of ECs gradually increased with the prolongation of ox-LDL treatment and the treatment of increased dose. The overexpression of LINC00460 enhanced the viability and EdU-positive rate in ECs treated with ox-LDL. miRNA-24-3p was the direct target of LINC00460, which was negatively regulated by LINC00460. miRNA-24-3p was downregulated with the prolongation of ox-LDL treatment. The overexpression of miRNA-24-3p could reverse the effect of LINC00460 on regulating the proliferative ability of ECs.

**Conclusions:**

LINC00460 regulates the proliferative ability of ECs and thus the occurrence and development of coronary atherosclerotic diseases by targeting miRNA-24-3p.

## 1. Introduction

Cardiovascular and cerebrovascular diseases are resulted from hyperlipidemia, blood viscosity, atherosclerosis, and hypertension, leading to the ischemic or hemorrhagic lesions in the heart, brain, and whole body [1]. The causes of cardiovascular and cerebrovascular diseases mainly include atherosclerosis, hypertension, hyperlipidemia, diabetes, leukemia, thrombocytosis, and other factors [2]. Cardiovascular and cerebrovascular diseases seriously threaten the human health, especially middle-aged and elder people over 50 years. They are characterized by high prevalence, disability, and mortality. There are 15 million people die of cardiovascular and cerebrovascular diseases every year, ranking the first in disease mortality [3]. Taking aspirin to prevent vascular embolism, changing lifestyles, and controlling blood pressure and blood lipids are considered to effectively prevent the cardiovascular and cerebrovascular diseases [4].

Endothelial cells exert a crucial role in the progression of cardiovascular diseases, such as coronary heart disease, stroke, and atherosclerosis [5]. Abnormal migration and proliferation of ECs owing to epigenetic changes are identified to participate in the pathogenesis of cardiovascular diseases [6]. Nevertheless, specific mechanisms underlying the abnormal cellular behaviors of ECs remain unclear.

Long noncoding RNAs (LncRNAs) are noncoding RNAs with over 200 nucleotides long [7, 8]. Numerous studies have shown the important role of noncoding RNAs in the epigenetic regulation [9]. LncRNAs could able to affect a variety of cellular processes [10]. Dysregulated lncRNAs are involved in many diseases, such as tumors, diabetes, and cardiovascular diseases [11]. Recent studies have found that LINC00460 promotes the development of nasopharyngeal carcinoma by absorbing microRNA-149-5p (miR-149-5p) to upregulate IL-6 [12]. LINC00460 regulates KDM2A by targeting miR-342-3p in gastric cancer and thereafter promotes proliferative and migratory abilities of cancer cells [13]. Upregulation of LINC00460 inhibits colorectal cancer to proliferate [14]. LINC00460 accelerates the progression of epithelial ovarian cancer by modulating miRNA-338-3p [15]. LINC00460 stimulates the malignant growth of lung adenocarcinoma by targeting the miR-302c-5p/FOXA1 axis [16]. The role of LINC00460 in the development of cardiovascular diseases, however, is poorly understood.

## 2. Materials and Methods

### 2.1. Cell Culture

ECs were provided by American Type Culture Collection (ATCC) (Manassas, VA, USA) and cultured in Dulbecco's Modified Eagle Medium (DMEM) (Gibco, Rockville, MD, USA) containing 10% fetal bovine serum (FBS) (Gibco, Rockville, MD, USA), 100 *μ*g/mL penicillin, and 0.1 mg/mL streptomycin, in a 37°C and 5% CO_2_ incubator. ECs were treated with 100 *μ*g/mL ox-LDL to mimic the high-fat environment.

### 2.2. RNA Extraction and Quantitative Real-Time Polymerase Chain Reaction (qRT-PCR)

Cells were digested in TRIzol Reagent (Invitrogen, Carlsbad, CA, USA) to isolate total RNAs. RNAs were then reversely transcribed into complementary deoxyribose nucleic acid (cDNA) and subjected to PCR using the SYBR Green method (TaKaRa, Tokyo, Japan) for 5 min at 94°C and 40 cycles for 30 s at 94°C, 30 s at 55°C, and 90 s at 72°C. The primers are as follows, LINC00460, F:5′-AGAAATCCTCCAGCCCTGTT-3′, R:5′-GGGTGACTCTTAGCCGAGAA-3′; GAPDH, F:5′-GAAGAGAGAGACCCTCACGCTG-3′, R:5′-ACTGTGAGGAGGGGAGATTCAGT-3′.

### 2.3. Dual-Luciferase Reporter Gene Assay

ECs were cotransfected with miRNA-24-3p mimic/negative control and LINC00460-WT/LINC00460-MT, respectively. After transfection of 48 h, cells were lysed for determining relative luciferase activity.

### 2.4. Cell Counting Kit-8 (CCK-8)

ECs were seeded in the 96-well plate at 5 × 10^3^ cells per well and cultured overnight. Absorbance (*A*) at 450 nm was recorded at the appointed time points using the CCK-8 kit (Dojindo Laboratories, Kumamoto, Japan) for depicting the viability curves.

### 2.5. Construction of Overexpression Vectors and Transfection

After constructing the pcDNA3.0-LINC00460 vector based on amplification with specific primers, the cDNA of LINC00460 was cloned into the mammalian expression vector pcDNA3.0 (Invitrogen, Carlsbad, CA, USA). ECs were cultured until 60% of confluence and subjected to transfection using Lipofactamine 2000 (Invitrogen, Carlsbad, CA, USA). 6 hours later, complete medium was replaced. Transfected cells for 24-48 h were harvested for in vitro experiments.

### 2.6. 5-Ethynyl-2′-Deoxyuridine (EdU) Assay

ECs were seeded in the 96-well plate with 300 cells per well. ECs were labeled with 50 *μ*mol/L EdU (Guangzhou RiboBio Co., Ltd., Guangzhou, China) at 37°C for 2 h. After 30-min fixation in 4% paraformaldehyde, cells were incubated with phosphate buffered saline (PBS) containing 0.5% Triton-100 for 20 min. After washing with PBS containing 3% bovine serum albumin (BSA), cells were incubated in 100 *μ*L of dying solution in dark for 1 h and counterstained with 100 *μ*L of 4′,6-diamidino-2-phenylindole (DAPI) (5 *μ*g/mL) for 30 min. EdU-positive cells, DAPI-labeled nucleus, and merged images were captured under a microscope (magnification ×100).

### 2.7. Statistical Analysis

Statistical Product and Service Solutions (SPSS) 18.0 (SPSS Inc., Chicago, IL, USA) was used for data analyses. GraphPad Prism 6.0 (La Jolla, CA, USA) was applied for depicting figures. Data were expressed as mean ± standard deviation. Differences between two groups were compared by the *t*-test. Differences among multigroups were compared by ANOVA analysis. *P* < 0.05 is considered statistically significant.

## 3. Results

### 3.1. LINC00460 Was Upregulated after Ox-LDL Treatment

**Figure 1:**
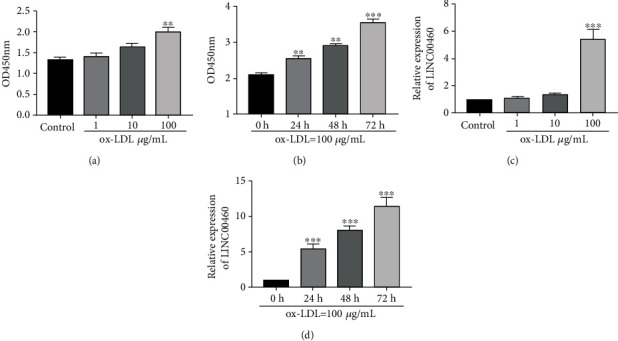
LINC00460 was upregulated after ox-LDL treatment. (a) CCK-8 assay showed the viability in ECs treated with 0, 1, 10, and 100 *μ*g/mL ox-LDL for 24 h. (b) CCK-8 assay showed the viability in ECs treated with 100 *μ*g/mL ox-LDL for 0, 24, 48, and 72 h. (c) Relative level of LINC00460 in ECs treated with 0, 1, 10, and 100 *μ*g/mL ox-LDL for 24 h. (d) Relative level of LINC00460 in ECs treated with 100 *μ*g/mL ox-LDL for 0, 24, 48, and 72 h.

Hyperlipidemia and abnormal lipid metabolism are important causes of atherosclerosis. The treatment of ox-LDL (oxidized low density lipoprotein) can aggravate atherosclerosis by stimulating the formation of foam cells, platelet adhesion and thrombosis, and EC injury. It is generally believed that ox-LDL is the important induction factor for atherosclerosis and in vitro EC injury. The viability of ECs was gradually enhanced with the treatment of increased dose of ox-LDL (Figure 1(a)). In addition, the viability increased with the prolongation of ox-LDL treatment (Figure 1(b)). QRT-PCR data showed that LINC00460 was greatly upregulated after 100 *μ*g/mL ox-LDL treatment for 24 h (Figure 1(c)). Its level gradually increased after 100 *μ*g/mL ox-LDL treatment for 24, 48, and 72 h (Figure 1(d)).

### 3.2. The overexpression of LINC00460 Accelerated the Proliferative Ability of ECs

**Figure 2:**
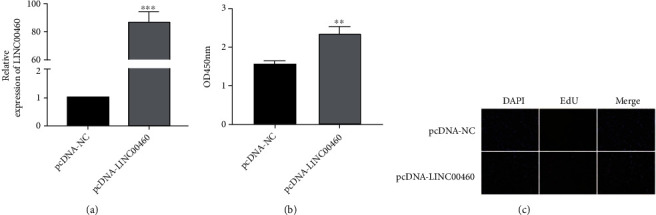
The overexpression of LINC00460 accelerated the proliferative ability of ECs. (a) Transfection efficacy of pcDNA-LINC00460 in ECs. (b) CCK-8 assay showed the viability in ECs transfected with pcDNA-NC or pcDNA-LINC00460. (c) DAPI-labeled, EdU-labeled, and merged images of ECs transfected with pcDNA-NC or pcDNA-LINC00460.

pcDNA-LINC00460 was constructed, and its transfection efficacy was tested. As qRT-PCR data revealed, transfection of pcDNA-LINC00460 remarkably upregulated LINC00460 level in ECs (Figure 2(a)). After transfection of pcDNA-LINC00460, the viability and EdU-labeled cells were enhanced, indicating the promoted proliferative ability of ECs (Figures 2(b) and 2(c)).

### 3.3. LINC00460 Bound to miRNA-24-3p

**Figure 3:**
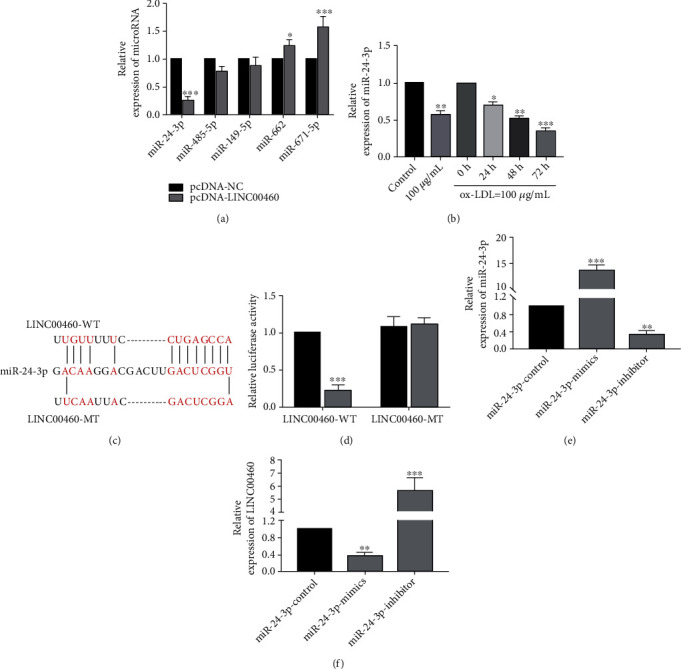
LINC00460 bound to miR-24-3p. (a) Relative levels of miRNA-24-3p, miRNA-485-5p, miRNA-149-5p, miRNA-662, and miRNA-671-5p in ECs transfected with pcDNA-NC or pcDNA-LINC00460. (b) MiR-24-3p level in ECs treated with 100 *μ*g/mL ox-LDL for 0, 24, 48, and 72 h. (c) Binding sites in promoter regions of LINC00460 and miR-24-3p. (d) Luciferase activity in ECs after cotransfection with miR-24-3p mimic/negative control and LINC00460-WT/LINC00460-MT, respectively. (e) Transfection efficacies of miR-24-3p mimics and miR-24-3p inhibitor. (f) LINC00460 level in ECs transfected with NC, miR-24-3p mimics, or miR-24-3p inhibitor.

Several potential miRNAs binding to LINC00460 were searched from online bioinformatic websites, including miRNA-24-3p, miRNA-485-5p, miRNA-149-5p, miRNA-662, and miRNA-671-5p. Transfection of pcDNA-LINC00460 downregulated miRNA-24-3p, but upregulated miRNA-662 and miRNA-671-5p (Figure 3(a)). After 100 *μ*g/mL ox-LDL treatment for 0, 24, 48, and 72 h, relative level of miRNA-24-3p gradually decreased (Figure 3(b)). Potential binding sequences between LINC00460 and miRNA-24-3p were identified in Figure 3(c). Subsequently, a remarkable decline in luciferase activity was observed after cotransfection of LINC00460-WT and miRNA-24-3p mimics, verifying the binding between LINC00460 and miRNA-24-3p (Figure 3(d)). To further uncover the biological function of miRNA-24-3p, we constructed miRNA-24-3p mimics and miRNA-24-3p inhibitor (Figure 3(e)). LINC00460 level was found to be negatively regulated by miRNA-24-3p (Figure 3(f)).

### 3.4. The Overexpression of miRNA-24-3p Reversed the Role of LINC00460 on EC Proliferation

**Figure 4:**
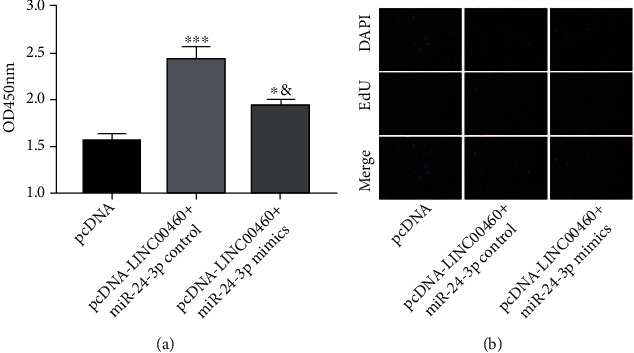
The overexpression of miR-24-3p reversed the role of LINC00460 on EC proliferation. ECs were transfected with pcDNA-NC, pcDNA-LINC00460 + miR-24-3p control, or pcDNA-LINC00460 + miR-24-3p mimics. (a) CCK-8 assay showed the viability. (b) DAPI-labeled, EdU-labeled, and merged images of ECs.

We further focused on the potential role of the LINC00460/miRNA-24-3p axis in influencing the proliferative ability of ECs. The elevated viability in EC overexpressing LINC00460 was reduced after cotransfection of miRNA-24-3p mimics (Figure 4(a)). Similarly, the number of EdU-positive cells was reduced after cotransfection of pcDNA-LINC00460 and miRNA-24-3p mimics compared with those overexpressing LINC00460 (Figure 4(b)). It is concluded that the miRNA-24-3p overexpression reversed the promotive effect of LINC00460 on the proliferative rate of ECs.

## 4. Discussion

Cardiovascular and cerebrovascular diseases are the leading causes of death in developed countries. Atherosclerosis is the main cause of coronary heart disease, cerebral infarction, and peripheral vascular disease [17]. Lipid metabolism disorder is the basis of atherosclerosis, which eventually leads to thickening and hardening of the arterial wall and narrowing of the vascular lumen [18]. Atherosclerosis is named because of the yellow atheroma appearance of deposited lipids in the intima [19]. The main risk factors for atherosclerosis include hypertension, hyperlipidemia, heavy smoking, obesity, and genetic factors. Symptoms of atherosclerosis mainly depend on the vascular lesions and the ischemic degree of the affected organs [20]. Severe complications of coronary atherosclerosis include angina pectoris, myocardial infarction, and even sudden death. Cerebral atherosclerosis may cause cerebral ischemia, brain atrophy, or cerebral vascular rupture [21]. Good living habits and discontinue the habit of smoking and drinking could prevent the occurrence of atherosclerosis [22].

MiRNAs are a class of noncoding, single-stranded RNAs encoded by endogenous genes. They are approximately 22 nucleotides in length and involved in the posttranscriptional regulation in plants and animals [23]. One miRNA regulates expressions of multiple genes, and several miRNAs could regulate the expression of a single gene. It is speculated that miRNAs are able to regulate one-third of human genes [24]. MiRNAs are capable of degrading mRNAs of the target genes through complementary base pairing in a complete way. Meanwhile, miRNAs inhibit the translation of target genes in an incomplete way without affecting the stability of the mRNAs [25]. Cellular behaviors could be mediated by miRNAs [26]. Abnormalities in miRNA expressions are involved in the disease development. For example, miRNA-210 induces the apoptosis of ECs by targeting PDK1 and thus aggravates atherosclerosis [27]. Exosomal-mediated miR-155 transfers it from smooth muscle cells to ECs and thereafter induces the occurrence of atherosclerosis [28]. Upregulated miRNA-876 induces the apoptosis of ECs by inhibiting Bcl-X1 [29]. This study mainly investigated the biological role of miRNAs in promoting proliferative ability of ECs.

Accumulating evidences have reported that lncRNAs act as miRNA sponges and eliminate the inhibitory effects of miRNAs on their target genes. For example, melatonin prevents the apoptosis of ECs by modulating the lncRNA MEG3/miR-223/NLRP3 axis [30]. The interaction of miR-193a-3p with HMGB1 suppresses ECs to proliferate and migrate [31]. LncRNA PVT1 activates CTGF/ANGPT2 by targeting miR-26b, thus promoting angiogenesis in vascular ECs [32]. SNHG15 affects the viability of glioma microvascular ECs through negatively mediating miR-153 level [33]. In this paper, we investigated the interaction between LINC00460 and miRNA-24-3p in influencing the proliferative ability of ECs.

The treatment of ox-LDL mimics the effects of atherosclerotic hyperlipidemia on ECs. LINC00460 was time-dependently and dose-dependently upregulated after ox-LDL treatment. The overexpression of LINC00460 enhanced the viability and EdU-positive rate in ECs treated with ox-LDL. Subsequently, dual-luciferase reporter gene assay verified the binding between LINC00460 and miRNA-24-3p. miRNA-24-3p was downregulated with the prolongation of ox-LDL treatment. Notably, the overexpression of miRNA-24-3p could reverse the regulatory role of LINC00460 on the proliferative ability of ECs. In this present study, we slightly uncover the role of LINC00460 in vascular endothelial cells by in vitro assay. However, the mechanism should be explored more deeply in the future research.

## 5. Conclusions

LINC00460 regulates the proliferative ability of ECs and thus the occurrence and development of coronary atherosclerotic diseases by targeting miRNA-24-3p.

## Data Availability

The datasets used and analyzed during the current study are available from the corresponding author on reasonable request.
